# Supplementary material for: A complex systems model of breast cancer etiology: The Paradigm II Model
Source: PLoS One. 2023 May 19;18(5):e0282878. doi: 10.1371/journal.pone.0282878 (PMC10198497; doi:10.1371/journal.pone.0282878)
Supplement: S1 Appendix — (DOCX) [file pone.0282878.s001.docx]

**Appendix**

We describe the branching process describing the number of cells of each class of development in each individual in the population. These describe both the joint probability distribution of the number of cells within each individual as well as the expected number of individuals with given cell counts in the population (conditional on the given covariates, discussed below).

Let *x* = [*x*_1_*,x*_2_*,...,x_N_*]*^T^* denote a vector of integer counts; *x_i_* represents the number of cells in class *i*, where *i* = 1*,...,N*. Here, *N* is the number of classes. We let *L_i_x* denote the lowering of index *i* by one cell, e.g., if *x* = [*x*_1_*,x*_2_*,...,x_N_*]*^T^*, then *L*_2_*x* = [*x*_1_*,x*_2_− 1*,...,x_N_*]*^T^*. Similarly, let *R_i_x* denote raising index *i* by one cell.

Let *u_x_*(*t*) be the probability that class 1 contains *x*_1_ cells, class 2 contains *x*_2_ cells, and so forth, at time *t* (*u_x_* represents the joint distribution of cell counts at all classes). If, for any index *i*, *x_i_ <* 0, then by

convention *u_x_* = 0.

We denote the per cell removal rate in class *i* by *ρ_i_*(*z*), where *z* represents individual-level covariates. The birth rate per cell in class *i* is denoted *b_i_*(*z*), and the rate of progression from class *i* to class *i*+1 is denoted *ν_i_*(*z*). In general, the individual-level covariates *z* represents the time-dependent action of environmental exposures and changes in estrogen levels over the life course, as well as other time-independent or dependent effects. We write *ρ_N_*_+1_ = 0 and *b*_−1_ = 0.

The model is specified by the Kolmogorov forward equations for the branching process at the individual

level:


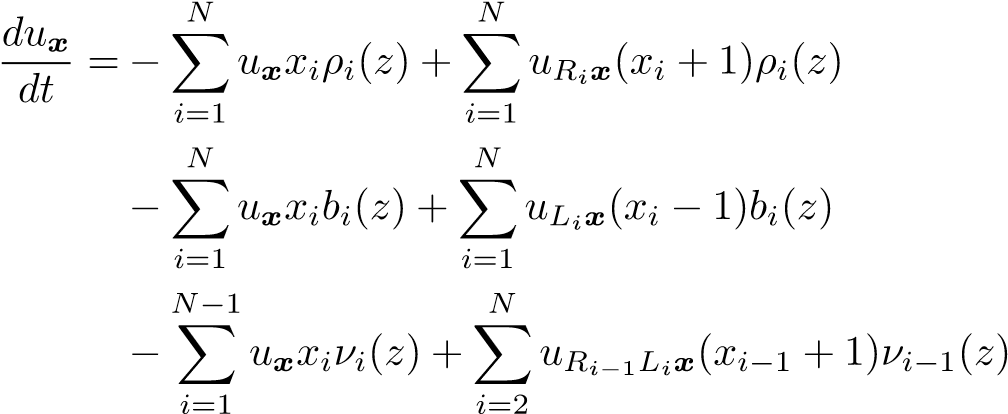
*.*

The first pair of terms on the right hand side represents loss or removal of cells from class *i*, the second pair represents growth within class *i*, and the final pair represents transitions from one class to the next.
