# Supplementary material for: A complex systems model of breast cancer etiology: The Paradigm II Model
Source: PLoS One. 2023 May 19;18(5):e0282878. doi: 10.1371/journal.pone.0282878 (PMC10198497; doi:10.1371/journal.pone.0282878)
Supplement: Attachment — Submitted filename: NOTES_PLoS One Reviuew.docx [file pone.0282878.s002.docx]

The authors should be commended for their ambitious and endeavor to create a complex systems population-oriented model of breast cancer incidence. Making a model like this represents a substantial undertaking, and the authors have wisely assembled an excellent transdisciplinary team. I believe the Paradigm II model has the potential to be a useful population health tool. However, I think that the current manuscript needs substantial work to achieve the goals of 1) understandability by a broad readership, 2) transparency, and 3) making a strong case that the models outputs are trustworthy. I provide a point-by-point critique below, but general themes include some ambiguous language in the Methods section, a lack of rationale for certain design choices, a lack of framing for how/why the results presented demonstrate the utility/credibility of the model, and a lack of explanations for discrepancies between modeled and observed results based on the same data sources. I believe that providing more methodological detail in one or more additional appendices would allow the authors to make the Methods section more readable while including the necessary detail needed for transparency/reproducibility.

Abstract

- Some empirical metrics in the Abstract seem in order.
- Line 59 – ‘The resulting model closely replicates most risk factor distributions’ does not seem capture the type of results presented. The results section of the paper presents mostly summaries of how the model reproduced incidence or relative risk due to specific risk factors.

Introduction

- Lines 123-126 – The placement of this language, and the phrasing, may suggest to some that the manuscript is going to address the enumerated questions. I think it is important to illustrate the rationale for developing the PII model, but I suggest rephrasing to signal to the reader that you are talking about future applications of the model.

Methods

- Generally, some detail is needed about the specific calibration approaches used.
- 140 – A bit of detail is needed about the ‘previously described methods’.
- 147 – The concept of ‘removal’ needs to be explained clearly early on.
- 161-162 – A reference is needed for this statement.
- 173- Drop first ‘incliuded’
- 180 – I found the sentence beginning at line 180 quite unclear.
- 191 – Does ‘healthy tissue’ refer to ‘healthy dense tissue’? Is non-dense tissue relevant to the events of interest in the model?
- 225-231 – Is total breast tissue relevant, or just dense breast tissue?
- 226 – What is meant by ‘others’? Does this refer to subsequent modified tissue sates?
- 238-240 – It Is said that both breast density and the ‘effects of breast density’ are a function of dense volume and BMI. Is there a distinction in meaning between these statements. If so, that distinction needs to be better clarified. If not, the redundancy needs to be eliminated.
- 240-242 – Is this statement not redundant of the statement in 235-237?
- 255-256 – it is a minor limitation that polygenic risk score was not conditioned on any other variables like race.
- 277 – The meaning of the phrase ‘risk data’ is not clear. Does this refer to cumulative incidence?
- 271-277 – The authors may want to briefly mention the rationale for directly considering only BRCA1 and not BRCS 2.
- 311-315 – I find this sentence confusing. Is one of the points that start/stop years were specified relative to menarche and menopause?
- ‘Stage’ is used to describe different model states stemming from cell damage in the early part of the paper (e.g. lines 154 and 191). Is ‘class’ as used on line 534 intended to convey the same concept? How does this concept different from ‘classes of damage’ used throughout the latter part of the paper?
- 353-358 – I found these sentences quite confusing. The rationale for choosing the particular rules for damage needs to be better explained. I think that a figure tied to the Methods section (perhaps a more detailed Figure 1) could help convey the concept of transitions and the types of damage relevant to each.
- 358-362 – Is this describing a calibration approach? Please clarify.

Results

- Figure 3 – The model seems to substantially overestimate incidence in women in their 70’s.
- Figure 13 – This figure is very difficult to read. Some more descriptive variable labels are needed for the horizontal (outcomes) axis. What are the units represented by the scale?
- 394-397 – Please explain the rationale for using simulated RCTs as the basis for analysis of OC, HT, and screening as an alternative to stratifying observed data from the synthetic population.
- III.B.3 / Table 1 – The same end points used to calibrate the PII model (Reference 53) appear to be used as comparators to the modeled outputs in Table 1. I am not sure of the value that this exercise provides, particularly given that the modeled and observed effect estimates do not correspond well.
- III.B.5 / Table 2 – A similar critique can be applied as for the authors’ treatment of oral contraceptive exposure (III.B.3 / Table 1) discussed above.
- III.B.9 / Table 3 – A similar critique can be applied as for the authors’ treatment of oral contraceptive exposure (III.B.3 / Table 1) discussed above.

Discussion

- 486-488 – I think it would be helpful to the reader in understanding the ‘big picture’ to bring out this point more strongly in the introduction.
- Generally, I think it would be helpful to provide some pros and cons of PII relative to the CISNET models of breast cancer incidence and screening.
