# Supplementary material for: A complex systems model of breast cancer etiology: The Paradigm II Model
Source: PLoS One. 2023 May 19;18(5):e0282878. doi: 10.1371/journal.pone.0282878 (PMC10198497; doi:10.1371/journal.pone.0282878)
Supplement: Attachment — Submitted filename: NOTES_PLoS ONE Re-Review.docx [file pone.0282878.s003.docx]

Authors: Thank you for addressing these critiques so thoroughly. I feel the revised version provides the clarity that this important work deserves.

Johnie Rose, MD, PhD

**Please address the following:**

107: With regard to the statement, “‘but do not inform interventions and policy in population health”, I don’t think this statement can be applied to CISNET given its role in informing screening recommendations.

153 – “93,826,718 women in the state of California in 2008-2012” – Is this female person-years? The population of CA is only ~40 million. Please clarify.

242-244: ‘the size of the healthy pool of breast tissue was assigned as a constant multiple of the amount of dense breast tissue with the multiplier calibrated to yield the needed incidence pattern.’ Is this to say ‘‘at time 0”? If so, this should be clarified.

396 – Please elaborate on the definition of “slightly”.

495: Change ‘an’ to ‘a’

**Please consider the following changes:**

Perhaps be bolder with touting what’s special about Paradigm II! In the Introduction and Discussion, I think the unique value of a complexity approach could be drawn out a bit more. As pointed out in the paper, it can be used to test population health interventions in silico. I think it can be more strongly emphasized that this approach lends itself to understanding relative and synergistic contributions of inputs at multiple levels. Finally, I think it could be mentioned in the Discussion that the approach can provide a framework to understand the areas where more empirical data are needed.

359-360: “Individuals with a value of 1 for this variable were assumed to be smokers throughout their life-course”. In the Discussion, this could be cited as a specific limitation related to simplification. Adjusting this assumption to assume that some fraction of ever smokers were not lifetime smokers could be a useful exercise (for the future).

378 – Is there a better term than ‘treated’? ‘Experimental’?

515 – I would suggest mentioning the hypothesized reason for overestimating incidence in women over 75 here (small sample size), in the Discussion section, rather than in Results.
